# Viscoelastic Transition and Yield Strain of the Folded Protein

**DOI:** 10.1371/journal.pone.0028097

**Published:** 2011-12-08

**Authors:** Yong Wang, Giovanni Zocchi

**Affiliations:** Department of Physics and Astronomy, University of California Los Angeles, Los Angeles, California, United States of America; University of Manchester, United Kingdom

## Abstract

For proteins, the mechanical properties of the folded state are directly related to function, which generally entails conformational motion. Through sub-Angstrom resolution measurements of the AC mechanical susceptibility of a globular protein we describe a new fundamental materials property of the folded state. For increasing amplitude of the forcing, there is a reversible transition from elastic to viscoelastic response. At fixed frequency, the amplitude of the deformation is piecewise linear in the force, with different slopes in the elastic and viscoelastic regimes. Effectively, the protein softens beyond a yield point defined by this transition. We propose that ligand induced conformational changes generally operate in this viscoelastic regime, and that this is a universal property of the folded state.

## Introduction

Solids have a shape while liquids flow. This is the situation for simple materials at low stresses, but for complex (e.g. composite) materials, or large stresses (e.g. plastic deformations) the behavior can be in between. Subjected to mechanical forcing (e.g. a shear), a material might be elastic and store mechanical energy (e.g. solids), viscous and dissipate mechanical energy (e.g. fluids), viscoelastic and both store and dissipate mechanical energy (e.g. complex fluids), plastic, viscoplastic, etc. Viscoelastic materials include polymeric solutions and melts, concentrated suspensions, and composites such as cells and tissue. For linear viscoelasticity, the rheological properties can be described in terms of the complex elastic modulus 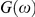 which gives the stress 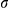 induced in the material by an applied oscillatory strain *ε* at frequency 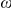: 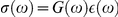. The real part of the complex modulus, 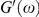 (also called the storage modulus), parameterizes the elastic response, while the imaginary part, 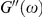 (also called the loss modulus), describes the viscous response. With *ε* 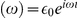 we see that for example for purely elastic behavior (the stress is proportional to the strain) at low frequencies (inertial effects are negligible; the stress is in phase with the strain) 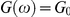 where 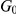 is a real constant, while for purely viscous behavior (the stress is proportional to the strain rate 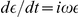) 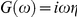 where 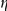 is real, i.e. 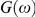 is pure imaginary.

The folded state of proteins is a peculiar material with some attributes of a crystal (e.g. a unique ground state) and some attributes of an amorphous solid (e.g. the lack of translational symmetry). The mechanical properties are central to the function, as ligand binding, catalysis, and allosteric regulation all involve conformational motion, i.e. deformations of the structure. However, while structural studies of conformational transitions abound, mechanical studies on the folded state are very limited, because of a lack of experimental means. Rms fluctuations measured in elastic scattering experiments yield zero frequency values of the elastic constants [1], [2], while force spectroscopy experiments [3], [4] with micro-mechanical methods such as the AFM probe the dynamics of unbinding, unfolding, and viscous dissipation in the unfolded state [3]–[12]. We have recently introduced a nano-rheology technique which exploits sub-Angstrom resolution to explore the mechanical properties of the folded state of proteins [13]. We found a transition from elastic behavior at low forcing amplitudes to viscoelastic behavior at higher forcing [14]. The purpose of this paper is to characterize this transition and the viscoelastic regime. We find that the force vs deformation is piecewise linear at fixed frequency: beyond a critical force (qualitatively analogous to a yield stress in macroscopic materials) the protein softens. We further show that within the simplest (Maxwell) model of viscoelasticity the force vs deformation curve reported here is quantitatively consistent with our previous measurements in the frequency domain [14]. Finally, we speculate that this viscoelastic transition is a universal mechanical property of the folded state, and that it is relevant for the large conformational changes which often accompany substrate binding in proteins.

### Nano-rheology of the folded state

**Figure 1:**
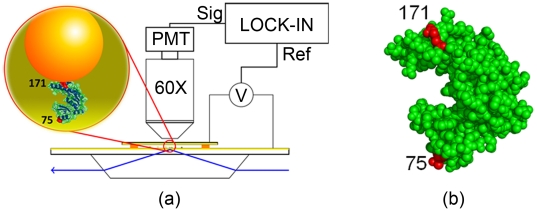
Experimental setup. (a) Schematics of the experimental setup including the chamber, electric excitation, and optical readout. The inset shows the geometry of the protein (Guanylate Kinase, GK) attached by the 171 and 75 sites to a gold nanoparticle (GNP) and a gold film evaporated on the glass slide (not to scale). (b) Crystal structure of GK (PDB: 1S4Q) with the attachment sites 75 and 171 highlighted.

The experimental system consists of a layer of 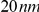 diameter gold nanoparticles (GNPs) tethered to a gold surface through the protein under study (Fig. 1). The surface is a gold film (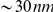 thick) evaporated on a glass slide, which serves both to anchor the proteins (through the SH group of specifically introduced Cysteins) and as a semi-transparent electrode. A 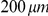 thick flow cell is constructed with this slide and a similarly gold coated cover slip, in a parallel plates capacitor configuration. An AC voltage applied to these electrodes drives the GNPs through the electrophoretic force, the GNPs carrying a large negative charge due to surface bound charged polymers (ss DNA 32mers). The “vertical” (perpendicular to the surface) motion of the GNPs, averaged over a large ensemble of GNPs (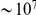), is detected by evanescent wave scattering [15], the signal being recovered at the forcing frequency in a phase locked loop. The combination of noise rejection due to the synchronous detection and averaging over many particles makes it possible to measure the ensemble average amplitude of oscillation of the GNPs with sub-Angstrom resolution (see Figure S1, Figure S2, Figure S3, and Figure S4).

The protein of this study is Guanylate Kinase (GK) from 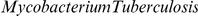; we [16], [17] and others [18] have been exploring its mechano-chemical properties with different methods over the past few years. GK is an essential enzyme which catalyzes the transfer of a phosphate group from ATP to GMP. The substrate binds in the cleft between the two lobes of the structure (Fig. 1b); GMP binding is of the induced fit type [18]–[21], the two lobes closing on the substrates through a 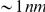 conformational change. The specific molecule of this study is the 75/171 mutant of [16], where Cys have been substituted at positions 75 and 171 (Fig. 1b); through these Cys the enzyme is anchored to the gold surfaces. We know from previous studies [16], [21], [22] that a mechanical stress in the 75–171 direction couples to the enzymatic function, specifically the binding affinity for GMP can be modulated through such stresses. A beautifully detailed representation of the mechanics of this enzyme is given in the simulations of the Baaden group [18], [23].

**Figure 2:**
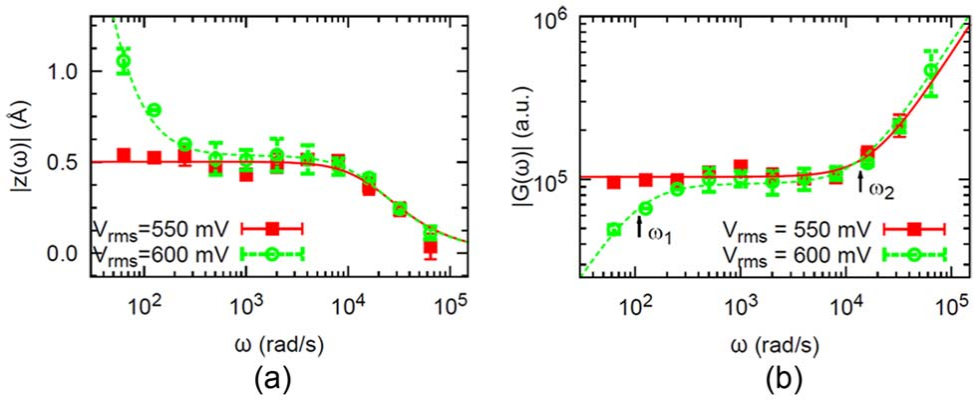
AC susceptibility of GK (from[**14**]). (a) Amplitude of the protein deformation vs frequency for two different amplitudes of the force (corresponding to driving voltages 550 and 600 mV). (b) Magnitude of the complex modulus 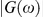 (arbitrary units) of the protein + GNP system; this is the same data as in (a) replotted as 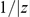 vs 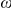. For 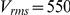 mV (squares) the behavior is elastic; for 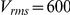 mV (circles) the behavior is viscoelastic. The error bars represent standard deviations of 5 measurements. The real “complex modulus” corresponding to 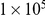 in the graph is 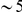 pN/nm. The points are experimental data while the lines are fits with the corresponding elastic or viscoelastic models.

For this system, we measured the response to a sinusoidal applied force in the frequency range 10 Hz–10 kHz [13], [14]. Below we summarize our previous results as they are relevant for what follows. For low amplitude of the force, the response (the amplitude of oscillation of the GNPs at the forcing frequency, averaged over many GNPs) is given by the squares in Fig. 2a. This is the response of a mass-less damped spring (continuous line in Fig. 2a), exhibiting a corner frequency 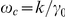 where 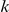 is the spring constant and 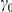 the dissipation coefficient. The low frequency plateau (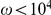) is due to the elastic constant of the protein (represented by 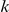), the high frequency cutoff (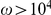) is due to the hydrodynamic dissipation of the GNP (represented by 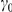). We have shown through these measurements that when the enzyme binds the substrate GMP, it stiffens by 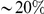, i.e. 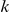 increases by 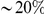 with a corresponding increase in 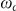 while 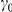 remains the same [13]. Since the enzyme is able to selectively bind its substrates, it is presumably in the folded state. This is confirmed by measurements of the enzymatic activity of the surface-immobilized enzyme (see Figure S5).

For larger amplitude of the force there is a transition to a qualitatively different response, given by the circles in Fig. 2a. The amplitude “diverges” at low frequencies. This is the response of a viscoelastic element (a spring and dashpot in series) attached to the GNP (dotted line in Fig. 2a), exhibiting two characteristic frequencies 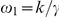, 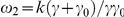 where 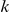 is the spring constant, 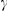 the dissipation coefficient of the dashpot (representing internal dissipation in the protein), 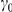 the hydrodynamic dissipation coefficient of the GNP [14]. The increase of the response amplitude for 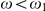 is the signature of viscoelasticity; the system “flows” at low frequencies.

## Results

**Figure 3:**
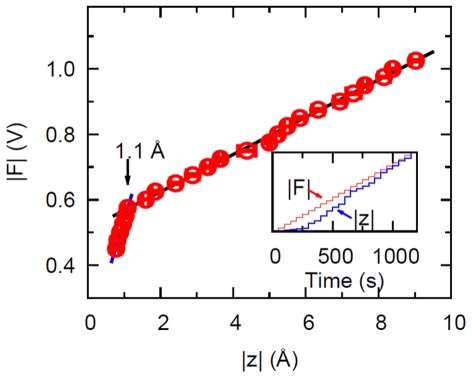
Dynamic force-extension curve. Force vs deformation measured at 10 Hz for the protein Guanylate Kinase, exhibiting a yield deformation of 1.1 Å. The force is in arbitrary units (corresponding to the voltage applied to the chamber), the deformation in Å. The error bars represent the standard deviation of 5 measurements. Inset: Stepwise increase of the applied force and the corresponding averaged amplitude of the protein's deformation in real time.

**Figure 4:**
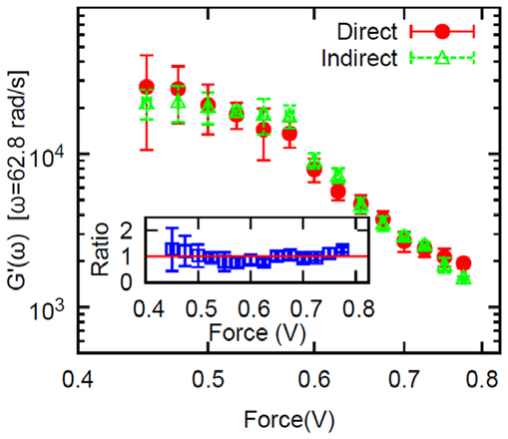
Direct measurement of the storage modulus. Circles: the storage modulus of the folded protein vs applied force measured directly at 10 Hz. Triangles: the storage modulus calculated indirectly from the magnitude of the complex modulus (i.e. from the data shown in Fig. 2b and using eq. (18)). Inset: the ratio (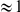) of the two modulii vs driving force. The error bars for 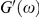 represent the standard deviation of 5 measurements.

We have shown in [14] that with increasing driving force the folded state of the protein undergoes a reversible transition from the elastic regime to a viscoelastic regime. Here we investigate this transition in detail, through “dynamic stretching” experiments where the frequency of the AC driving force is kept constant while the amplitude is varied. This corresponds to moving along a vertical line in the graph of Fig. 2a, where the control parameter is the amplitude of the force. We fixed the driving frequency at 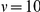 Hz (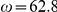 rad/s) where the difference in response between the elastic and viscoelastic regimes is large (Fig. 2). The forcing amplitude is increased in steps, with 50 s waiting times between steps; correspondingly, the response amplitude also increases in steps (inset of Fig. 3). In Fig. 3 we plot the “dynamic force-extension curve”, i.e. the amplitude of the driving force versus the amplitude of the response (averaged over the 50 s waiting time). This dynamic force-extension curve is piecewise linear: the break at 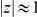 Å is the reversible transition to the viscoelastic regime. Thus there is a yield strain (here 1 Å/40 Å = 2.5%) beyond which the protein “softens”, while maintaining a linear relation between force and deformation. We also note that the value of the yield strain must be frequency-dependent. The transition from the elastic (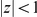 Å) to the viscoelastic (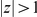 Å) regime is reversible (the same piecewise linear curve can be repeated for the same sample as a function of the driving amplitude), but not if one exceeds a certain driving voltage (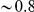 V in these experiments) in which case turning the driving down does not reproduce the same states. In the next section we show that the measured response vs frequency (Fig. 2a) and the measured response vs force (Fig. 4) are quantitatively consistent with each other when interpreted in terms of a transition of the system from elastic to viscoelastic behavior.

### Complex Modulus formalism and Maxwell model

We start with the generalized linear relation between force and displacement: [24], [25], 
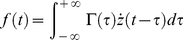
(1)


where 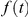 is the applied force, 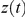 is the displacement, and 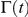 is the “relaxation modulus”, implicitly satisfying the causality condition 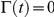 if 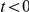. In the frequency domain, 
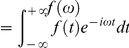
, 
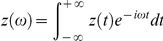
, we have the generalized Stokes Einstein relation (GSER) [26]–[28], 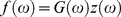
(2)


where 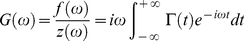
(3)


Here 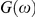 is a “complex spring constant”, but we find this term awkward so we will borrow a term from rheology instead and call our 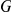 the “complex modulus”. In the rheological literature the relations above are written for the stress and strain; the function 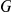 (the complex modulus) has then dimensions of a force per unit area. Here we choose to use force and displacement, instead of stress and strain, to describe the system for the reason that force and displacement are operationally well-defined in our experiments. In contrast, stress and strain are properly defined over length scales which are large compared to the atomic structure, but small compared to the macroscopic volume of the material under consideration [24]. Thus it is questionable whether stress and strain are well defined quantities for a single protein molecule. On the other hand, the relation (1) just expresses a general linear relationship, which here we assume between force and displacement. From (1) and (2) 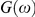 has then dimensions of a force per unit length. In analogy with the rheological terminology, where the real and imaginary parts of the complex modulus are often called the storage and loss modulus, respectively, in the following we call the real and imaginary part of our 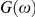 the same.

Let us form a qualitative picture of what to expect for the measurements of the complex modulus of the protein, 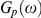. In the elastic regime (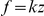) the “complex modulus” is simply a real constant: 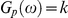. The eigenfrequencies of the protein lie in the range above 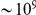 Hz [29]; this is also easily estimated from a typical “spring constant” 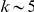 pN/nm [30] and the mass of the protein 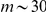 kDa which gives a fundamental mode 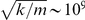 Hz, or equivalently from a typical Young's modulus 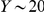 MPa, density 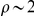 g/cm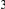, and size 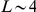 nm, giving 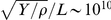 Hz. Therefore in the frequency range of the experiments, 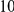 Hz 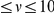 kHz, in the elastic regime one expects to see the low frequency response of a spring which corresponds to a constant complex modulus.

In the experiments, the protein is attached to a Gold nanoparticle (GNP). The equation of motion of the GNP is [13], [14]: 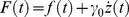
(4)


where 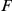 is the electrophoretic force applied to the GNP, 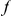 the force on the protein, 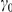 the hydrodynamic dissipation coefficient of the GNP (because of the proximity of the surface, 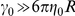, the Stokes drag coefficient, where 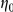 is the viscosity of the fluid and 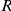 the radius of the GNP). The inertial term has been neglected in eq. (4) because in the experiments the driving frequency is “small”: 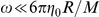 where 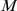 is the mass of the GNP. Also, there is no Brownian motion term because the measurement method averages it out (this is the reason why with the present method one can measure displacement amplitudes of a fraction of 1 Å, see Figure S1, Figure S2, Figure S3 and Figure S4).

We wish to show that the measured response of the system is consistent with a viscoelastic transition of the protein [14]. The simplest model of viscoelasticity is the Maxwell element, which is a spring and dashpot in series. The corresponding equation of motion 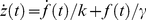
(5)


(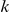 is the stiffness of the spring, 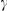 the dissipation coefficient of the dashpot, 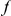 the applied force, 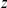 the displacement) gives a complex modulus 
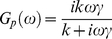
(6)


where 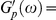 Re 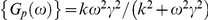 is the storage modulus and 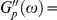
Im 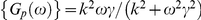 is the loss modulus. From eqs. (5) and (4) we obtain [14]: 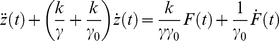
(7)


With a sinusoidal force of amplitude 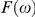: 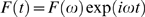 the response is an oscillation of amplitude 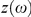: 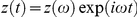 (with the usual convention of taking real parts to obtain the physical quantities); substituting in (7) we obtain the complex modulus of the Maxwell element + GNP system (the protein + GNP system if the protein behaves like a Maxwell element): 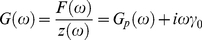
(8)


where 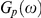 is given by (6). The following remarks will be useful in understanding the arguments below. First, a moment's reflection shows that the decomposition (8) is valid independently of the specific model assumed for 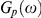, i.e. the contribution from the GNP to the complex modulus is purely imaginary, 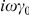. Therefore the storage modulus of the protein is in fact exactly the same as that of the protein 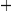 GNP system. Second, in the frequency response experiments, the amplitude of the driving force is kept constant at different frequencies, 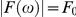. Then 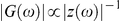 (see eq. (8)), i.e., the magnitude of the complex modulus is inversely proportional to the amplitude of the protein deformation 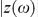, which is the quantity measured in the experiments. Therefore we may plot the experimental measurements either as 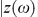 vs 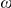, as in Fig. 2a, or equivalently as 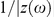 vs 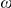, which is, apart from a multiplicative constant, the same as 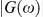 vs 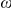. This is the way the experimental data are plotted in Fig. 2b. In conclusion, Fig. 2b shows the magnitude of the complex modulus vs frequency measured for GK for two values of the forcing amplitude. These are the same data as in [14], presented in terms of 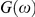. A transition between two different behaviors is apparent. For low forcing amplitude (squares), we get the response of a spring: the solid line is a fit using eq. (8) with 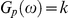, which is 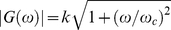
(9)


where 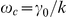. The increase of 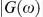 at high frequency (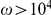 rad/s) reflects the hydrodynamic dissipation of the GNP (i.e. 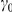). At higher forcing amplitudes (circles) we get the response of a viscoelastic element: the solid line is a fit using eq. (8) with 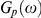 given by (6). This form is 
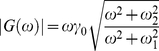
(10)


where 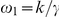, 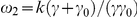. The fits give the values: 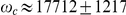 rad/s, 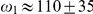 rad/s, 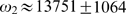 rad/s [14]. Since 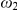 and 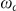 are essentially the same, 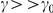 i.e. 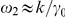 and 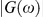 in the viscoelastic regime can also be written as: 
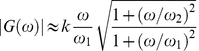
(11)


The drop of 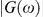 at low frequency is the signature of viscoelasticity. This transition from elastic to viscoelastic behavior is reversible: turning up the driving force the sample jumps from the elastic to the viscoelastic behavior, and turning the driving force down to the original value the same sample reverts to the elastic behavior [14].

We can now show that the piecewise linear response of Fig. 3 is quantitatively consistent with the measurements in the frequency domain Fig. 2, in the framework of the Maxwell model (6). Namely, the formulas (9), (11) read for the response amplitude in the elastic and viscoelastic regimes: 
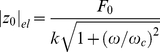
(12)




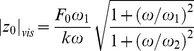
(13)


At fixed driving frequency (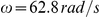 for the data of Fig. 3) the amplitude of the driving force is proportional to the amplitude of the protein deformation in both regimes. But the proportionality constants, or the slopes, are different in the two regimes. In the elastic regime, the slope is 
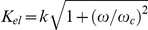
; while in the viscoelastic regime the slope is 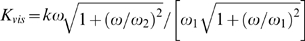. The ratio of the slopes is: 
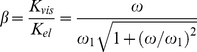
(14)


and using the value 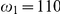 rad/s measured in the frequency response experiments (Fig. 2) and 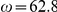 rad/s we obtain 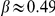. On the other hand, from the linear fits in Fig. 3 we can measure directly the ratio of the slopes and obtain 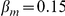. This shows a remarkable consistency of the two measurements Fig. 3 and Fig. 2 in the framework of the simplest viscoelastic model. We may also say that the ratio of the two slopes 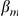 measured from Fig. 3 provides another way to estimate the internal friction (and thus the internal viscosity) of the protein, by assuming (14) and using the spring constant for the protein reported previously [30], 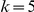 pN/nm. In this way we obtain the internal friction coefficient 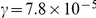 kg/s (or an internal viscosity of the protein 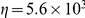 Pa

s), which is close to the value we obtained in [13] from the characteristic frequencies 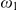, 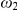. In summary, the ratio of the slopes in Fig. 3, which is about 6, is consistent with the characteristic frequency 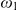 at which the response departs from simple elasticity (Fig. 2).

Given the nonlinear (piecewise linear) response displayed in Fig. 4, the question arises whether such nonlinearity alone, in the absence of internal dissipation, can give rise to the frequency response of Fig. 2, or in other words, is this a viscoelastic system or a nonlinear, non-dissipative system. The question can be answered (in favor of viscoelasticity) by numerically computing the frequency response of a nonlinear spring such as the one of Fig. 4. We do this in Figure S6. The result is that the piecewise linear response displayed in Fig. 3 cannot by itself, in the absence of internal dissipation in the protein, give rise to the frequency response displayed in Figs. 2. Instead, the experimental measurements show that the force vs displacement response of the protein is piecewise linear, corresponding to a transition from elasticity (where no internal dissipation of the protein is seen) to viscoelasticity, where there is internal dissipation and the protein is mechanically softer. This transition happens, in this case (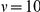 Hz), for a yield deformation 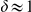 Å corresponding to a yield strain 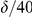 Å 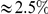.

If the deformation is not too small it is possible to measure directly in the experiments the real and imaginary parts of the deformation 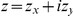 at a fixed frequency. Then the storage modulus is 
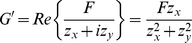
(15)


and represents the storage modulus (real part of the complex modulus) of the protein only, with no contribution from the GNP (see eq. (8)). The measurements are shown in Fig. 4, vs amplitude of the applied force. We now show that the measurements of 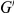 displayed in Fig. 4 are consistent with the measurements of 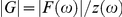 of Fig. 3, if a viscoelastic model is assumed. Because the frequency of these measurements is so low (10 Hz), the complex modulus 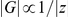 obtained from the data of Fig. 2 reports on the complex modulus of the protein, the contribution from the gold nanoparticles being negligible. In the viscoelastic regime, assuming the response is that of a Maxwell element, the real and imaginary parts of the complex modulus 
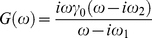
(16)

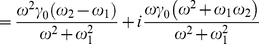
(17)


can be written in terms of 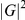 as: 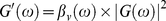
(18)

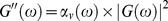
(19)


where 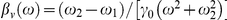, 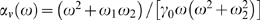, 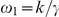 and 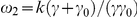. Similarly for the elastic regime: 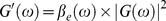
(20)

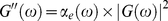
(21)


where 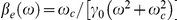 and 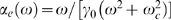 (see Figure S7).

The relation (18) for the storage modulus is somewhat model independent, in the sense that 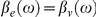 if 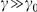, which is true in our experiments [10], [14]. The triangles in Fig. 4 are computed from the measurements of 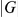 of Fig. 2, using the relation (18) to obtain 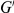 (normalized by a multiplicative constant). The signal over noise of the measurement obviously improves at larger forcing amplitudes, so particularly the direct measurements have larger error bars at low frequency (the error of the storage modulus 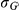 is estimated through error propagation 
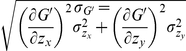
 where 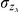 and 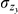 are the standard deviation of five measurements of 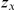 and 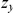, respectively); nonetheless Fig. 4 shows that no systematic deviation is seen between the storage modulus measured directly and computed from the measured 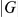 assuming the response of a Maxwell model. The inset in Fig. 4 shows the ratio between the two, the red line indicating a ratio of 1.

**Figure 5:**
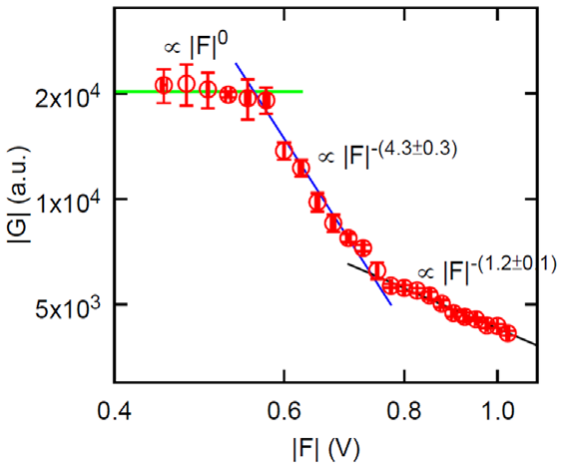
Complex modulus from the dynamic stretching experiments. Magnitude of the complex modulus of the protein as a function of the amplitude of the applied force. The error bars represent standard deviation of 5 measurements.

We mentioned that the transition from the elastic (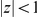 Å) to the viscoelastic (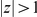 Å) regime is reversible (the same piecewise linear curve can be repeated for the same sample as a function of the driving amplitude), but not if one exceeds a certain driving voltage (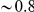 V in these experiments). There is indeed a second, irreversible transition, which is more apparent if we plot the same data of Fig. 3 in terms of the complex modulus 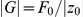 vs applied force 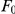, which is displayed in Fig. 5. There are three regimes: the elastic regime where 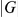 is a constant (independent of applied force), the viscoelastic regime which also entails a reversible, progressive softening of the protein with increased applied force, and finally an irreversible transition to a regime where the complex modulus also decreases with increased applied force, but slower than in the reversible viscoelastic regime. This graph also shows that not all properties of the system can be described by a linear model such as the Maxwell model, since in the viscoelastic regime the experimental 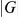 depends on the applied force. This is seen equivalently in Fig. 3, where the linear behavior in the viscoelastic regime does not extrapolate to the origin.

## Discussion

We present new measurements of the mechanical susceptibility of a folded protein. The sub-Angstrom resolution allows us to access both the elastic regime and what lies beyond. As the amplitude of the forcing is increased, we find a transition in the response of the protein which is displayed in Fig. 3. “To the left” of the transition (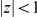 Å for 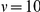 Hz) lies the elastic, non-dissipative regime. This is similar to any macroscopic solid such as a crystal, except that for the protein the elastic regime extends to considerably larger strains (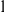 Å/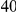 Å 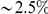) than for a regular solid, where yield strains for plastic deformations are typically 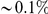. “To the right” of the transition (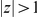 Å) lies a viscoelastic regime where internal dissipation in the protein is prominent. We have further shown that several (but not all) of the experimental measurements can be summarized or described in terms of a transition from the elastic to a viscoelastic regime. Specifically, for a fixed amplitude of the force the frequency response in the viscoelastic regime is described by the simplest (Maxwell) model of viscoelasticity. In terms of this model, there is a remarkable consistency between the two slopes of the piecewise linear force response Fig. 3 and the corner frequency 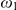 of the frequency response Fig. 2 (see eq. 14). Evidently this fact does not constitute a theory of the mechanical properties of the protein, but we find this way of summarizing the experimental results useful in that we can now make predictions. For example, if the “dynamic stretching” experiment of Fig. 3 is repeated at a higher frequency, (14) predicts that the slope in the viscoelastic regime should be correspondingly smaller (the slope in the elastic regime must remain the same). Experiments are under way to investigate this behavior.

In the viscoelastic regime, the force vs dis”placement curve is still linear (Fig. 3), however this is really a nonlinear viscoelastic regime, as can be appreciated from the representation of Fig. 5, which shows that in this regime the magnitude of the complex modulus decreases with applied force (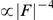 approximately); an analogous nonlinear phenomenon in complex fluids would be “shear thinning”. This signature of nonlinear viscoelasticity is of course also present in Fig. 3 (which shows the same data as Fig. 5, in a different representation), reflected in the fact that the straight line through the data in the viscoelastic regime does not extrapolate to the origin. It may be possible to represent this non-linearity by simply adding a force dependence to the parameter 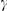 of the Maxwell model, but in the absence of a more fundamental theory it is not sure whether anything new would be learned. In the end, the system is nonlinear *and* viscoelastic, but we have further characterized the nonlinearity, namely, the force vs displacement curve is piecewise linear.

Finally, because the system is fundamentally non-linear (Fig. 3), the assumption (1) is in fact wrong. This does not mean that we cannot use (2) to define a useful function 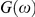, but we must remember that: 1). 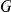 depends on the applied force; 2). this 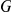 refers specifically to a sinusoidal applied force, and there is no guarantee that we can use it to calculate the response of the system to a different waveform of the perturbation (such as a step in the force), as we would be able to do for a linear system. We chose to discuss our measurements in the language of linear viscoelasticity because *for a fixed amplitude of the force* the frequency response of the system is simple: either elastic (for small enough force), or viscoelastic (for high enough force). The system is non-linear, but the non-linearity appears rather simple (Fig. 3).

We conjecture that the transition displayed in Fig. 3 is a universal property of the folded state of proteins, at least if the force is exerted in a direction which is not orthogonal (in some appropriate sense) to a functional conformational change of the protein (for the present system, GK undergoes a large conformational change in the 171/75 direction upon binding the substrate GMP). With only one system examined so far, this is obviously only a hypothesis, but it would be quite surprising if such a fundamental materials property, namely the way the molecule transitions beyond the elastic regime for large deformations, was completely different from protein to protein, as there are not too many possible different scenarios from a materials science perspective.

The transition from elastic to viscoelastic behavior happens (in this system and at this frequency) for a yield deformation (or critical deformation) 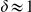 Å. Deformations characteristic of conformational changes associated with induced fit binding and allosteric transitions are often 10 times larger, of order 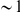 nm. For example, for the enzyme of this study (Guanylate Kinase) binding of the substrate GMP induces a conformational change in which several residues move about 1 nm relative to each other [19]. Therefore if the conjecture above is correct the typical functional conformational change in enzymes is a process operating across the transition described here into the viscoelastic regime. This viscoelastic transition then acquires a significance beyond the materials properties of the protein structure, as it affects the thermodynamics of the enzymatic cycle.

What is the microscopic picture of the viscoelastic transition? A deformation just beyond the yield strain 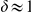 Å could correspond to breaking a single hydrogen bond for example, or it could be distributed over many bonds. This question (“distributed model” vs “bond model”) was introduced by Hopfield many years ago [31] in the context of cooperativity in hemoglobin, and is just as relevant here, but we do not have the answer. Another interesting question concerns the actual mechanism of internal dissipation in the viscoelastic regime: presumably bonds (e.g. hydrogen bonds) are broken and reformed in the process, exciting elastic modes in the protein which eventually couple to the water (literally!) bath; however, we do not know the precise mechanism, i.e. there is no first principles calculation of the dissipation coefficient 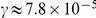 kg/s, which we measure in the experiments.

The phenomenology reported here - linear elasticity up to a yield stress beyond which the system is viscoelastic - is qualitatively similar to that observed in concentrated colloidal suspensions and colloidal crystals [32]. Perhaps this is not surprising: the folded protein *is* a close packed colloidal solid (at least for small enough deformations).

In conclusion, we document a viscoelastic transition of the globular protein, and suggest that it is a universal property of the folded state.

## Materials and Methods

### Materials

Gold nanoparticles (GNP, 20 nm diameter) were from Nanocs (New York, NY); other chemicals from Sigma-Aldrich. Experiments were performed in saline-sodium citrate buffer (SSC; Invitrogen) diluted with deionized water to a final concentration of 50 mM sodium chloride and 5 mM trisodium citrate, pH 7.0 (SSC/3).

### Sample Preparation

The protein under study (Guanylate kinase or GK, a 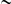
24 kDa, 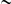
4 nm sized globule) was prepared by mutagenesis with the internal Cys changed to Ser and the residues at the positions 171 and 75 substituted by Cys, as described in [22]. The introduced Cys are essential for the protein to tether gold nanoparticles to a gold thin film on a glass slide (Fig. 1). The glass slide was thoroughly cleaned before evaporating a 3 nm layer of Cr followed by 30 nm of gold using an e-Beam vacuum evaporation system. The purpose of the gold layer is to obtain a conducting electrode (see details below) and also take advantage of the affinity of the thiol groups (Cys on the protein surface) for gold surfaces [33], [34]. In practice, the Au-slide was immersed in GK solution (2 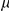
M in 1 M KH
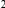
PO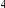 at pH 7.0) overnight and then washed with a large amount of deionized water. Gold nanoparticles (20 nm diameter) were then introduced and incubated at room temperature for 2 hours, followed by washing with water. In order to make the gold nanoparticles charged through surface modifications, and to remove nonspecific protein immobilization, the slide was then immersed in a solution of thiol-modified DNA (32 bases, 1 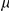
M in 1 M KH
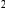
PO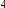 at pH 4.0) overnight and washed with water. The prepared slide forms the bottom of a chamber, which is constructed in a parallel capacitor configuration with a cover slip (also coated with a gold thin film) on top of the slide, separated by 200 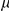
m spacers. After external electrodes are attached to the gold films, the chamber is filled with SSC buffer diluted by 3 and ready to use.

### Frequency Response Experiments

Frequency response experiments, which measure the AC susceptibility of the sample vs frequency, are described in detail in [13]. The experiment consists in mechanically forcing the gold nanoparticles using an AC electric field and detect their motion along the direction of the forcing by evanescent wave scattering in a phase locked loop (Fig. 1). The GNPs carry a large negative charge due to the DNA “brush” anchored to their surface, and thus can be driven by the electrophoretic force established by applying a potential difference across the chamber. The motion of the gold nanoparticles along the direction of the electric field, which is perpendicular to the gold surface, is monitored by evanescent wave scattering, where the displacement of the GNPs 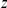 is proportional the change in the scattered light intensity 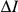, 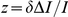, where 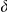 (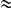
64nm in our setup) is the penetration depth and 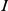 is the total light intensity scattered from a collection of gold nanoparticles (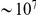 GNPs in a filed of view 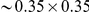 mm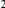). Therefore we detect the average displacement of this collection of GNPs, which reports on the average deformation of the tethered proteins. In the frequency response experiments, AC voltages at frequencies, 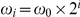 (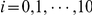 and 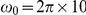 rad/s), are applied to the chamber and the displacements are measured in a phase locked loop, using a lock-in amplifier. At each frequency, the amplitude of the GNP displacement, called the “response”, is averaged over 50 seconds. The resultant averaged response as a function of the driving frequency, referred to as the “frequency response”, is then used to calculate the mechanical properties of the sample.

### Dynamic Stretching Experiments

Dynamic stretching experiments share the same setup (Fig. 1) with the frequency response experiments. The difference from the latter is that we now vary the amplitude of the driving AC voltage at a fixed driving frequency. In this study we chose 10 Hz as the driving frequency based on the observation that the difference in the response of the sample to driving forces between the elastic regime and the viscoelastic regime lies in the low frequency range [13] (and see “Results and Discussions” below). The amplitude of the AC voltage on the chamber is a linear series, ranging from 0.450 V to 1.025 V. At each amplitude, the response is measured and also averaged over 50 seconds, as in the frequency response experiments. By varying the amplitude of the applied AC voltage, a dynamic stretching experiment measures the relation between the amplitude of the driving force (proportional to the AC voltage) and the amplitude of the displacement, which we refer to as the “dynamic force-extension curve”.

## Supporting Information

Figure S1
**Thermal fluctuations are averaged out by measuring over many GNPs: case of an elastic tether.** Simulated displacement (a) of a single GNP; (b) averaged over 5000 GNPs, attached to elastic springs in the presence of thermal noise. We ran numerical simulations and looked at the displacement of GNPs attached to an elastic spring when a sinusoidal external force is applied. The equation of motion of the gold nanoparticle, including thermal fluctuations, is 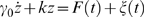 where 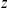 is the displacement of the GNP, 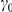 the hydrodynamic dissipation coefficient of the GNP, 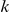 the spring constant, 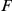 the applied external force: 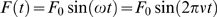, where 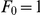 pN and the alternating frequency 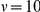 Hz, and 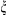 a stochastic force (the Brownian motion term) satisfying the following two relations: 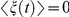 and 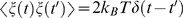. The parameters of the simulation were directly from experimental measurements [14], [30]: 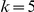 pN/nm, 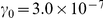 kg/s. We note that the applied force in the experiments is 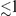 pN. In the presence of the Brownian noise term, the displacement of a single GNP is dominated by thermal fluctuations. However, the average displacement over 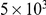 GNPs is not: the average displacement oscillates at the same frequency as the applied force. The simulation also shows that by averaging over many particles, it is not impossible to measure “very small” displacements (sub-Angstrom, at high frequencies, compared to thermal fluctuations of the individual GNPs are 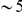 nm) buried in large thermal noise.(TIF)

Figure S2
**Thermal fluctuations are averaged out by measuring over many GNPs: case of a viscoelastic tether.** Simulated displacement (a) of a single GNP; (b) averaged over 5000 GNPs, attached to viscoelastic Maxwell elements (eq. 5) in the presence of thermal noise. We ran numerical simulations similar to the ones in Fig. S1 but with a slightly different equation of motion for the gold nanoparticle: 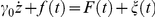 where 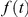 is the force from the Maxwell element (eq. 5). The parameters were again directly from experimental measurements [14], [30]: 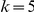 pN/nm, 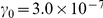 kg/s, 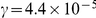 kg/s. Similar to Fig. S1, in the presence of the Brownian noise term, the displacement of a single GNP is dominated by thermal fluctuations. However, the average displacement over 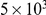 GNPs is not: the average displacement oscillates at the same frequency as the applied force. The simulation also shows that by averaging over many particles, it is not impossible to measure “very small” displacements buried in large thermal noise.(TIF)

Figure S3
**Simulated frequency response in the presence of thermal noise.** Fitting the average displacement (Fig. S1b and Fig. S2b) with a sine wave gives the amplitude and phase (the quantities we measure in the experiments). By sweeping the driving frequency (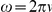) over a range, we obtain numerically the frequency response (a: amplitude; b: phase) in the presence of thermal noise, which are exactly the same as the analytical results from eq. (4), without the Brownian motion term. Thus the present measurement method is able to average out thermal noise.(TIF)

Figure S4
**AC susceptibility of a single stranded DNA (experimental measurements).** In addition to numerical simulations, we also show experimentally that the average displacement of many gold nanoparticles is sinusoidal with the same frequency as the driving force. In order to observe directly the oscillation of the average displacement, a single stranded DNA coil [13], [14], which is softer than a globular protein and has larger deformation, has been chosen; and also the driving frequency is low: 0.1 Hz. (a) The instantaneous displacement (thin black curve) averaged over 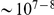 GNPs and a fit with a sine wave (thick red curve) giving a fitted frequency of 0.1 Hz, which is the same as the driving frequency. (b) Fourier transform (FFT) performed on the instantaneous displacement. An obvious peak is present at 0.1 Hz.(TIF)

Figure S5
**Enzymatic Activity of Immobilized Proteins.** We are interested in the mechanical properties of the *folded* protein. It is then essential to make sure that the proteins immobilized on the gold surfaces are folded and functional. We measured the enzymatic activity of GK attached to the Gold-coated slide using the Kinase-Glo (Promega, Madison, WI) luminescent assay. The assay quantifies the depletion of ATP following the kinase reaction: GMP+ATP
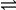
GDP+ADP. The assay reagents rely on the properties of a proprietary thermostable luciferase that is formulated to generate a stable “glow-type” luminescent signal which is produced by the luciferase reaction. The intensity of the generated luminescence is directly proportional to the amount (or concentration in a fixed volume) of ATP in the solution (see manufacturer manual). A mixture of ATP and GMP solution at optimized concentrations is added on the surface with the immobilized proteins. The kinase reaction is incubated for 2.5 or 4 hours. Then the solution is removed, mixed with the luminescence assay reagents and incubated for 10 minutes. Luminescence was measured with a DTX 800 multimode detector (Beckman Coulter). The figure shows the concentration of ATP remaining after the specified time for specifically immobilized guanylate kinase (+icrGK2075171: the mutant of this study, with Cys residues substituted at positions 75 and 171) and two controls: without proteins on the slide (Blank) and with nonspecifically bound GK (+icrGK: this mutant has no Cysteins). The result shows that the specifically immobilized Guanylate Kinase is functional and therefore folded on the gold surface.(TIF)

Figure S6
**Numerical simulation of nonlinear springs.** We computed numerically the frequency response of a nonlinear spring, with a force-extension curve: 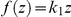 if 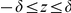, or 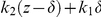 if 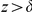, or 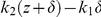 if 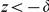, attached to a “bead” characterized by a hydrodynamic dissipation coefficient 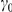 (eq. 4). In eq. (25), 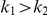 represent the two slopes of Fig. 3 and 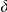 is the “yield deformation”. We ran the simulation using the measured values for the three parameters: 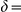 0.5 Å (Fig. 2), 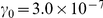 kg/s, 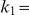
5 pN/nm [13], [30], and varying 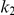 and the amplitude of the forcing. The figure shows the results for the amplitude of the response, for 4 different values of 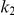 (and thus the ratio 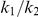; 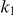 is fixed at the value of the elastic spring constant of the protein) under different driving forces: 0.25, 0.26 and 0.27 pN. The figure shows that one can obtain a response similar to the inset in Fig. 2, but only for very large ratios of the slopes 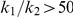. In contrast, the experimentally measured ratio (Fig. 3) is 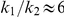, and for such “nonlinear springs” the response always looks like the last graph, quantitatively and qualitatively different from Fig. 2. Nonlinear springs with small 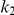
's show Maxwell-type response. But a nonlinear spring with large 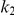 does not.(TIF)

Figure S7
**Calculated real and imaginary parts of the complex modulus.** We represent the same data and fits of Fig. 2 in terms of storage modulus 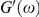 and loss modulus 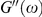 calculated from (eqs. 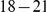), since this is the canonical representation in the rheological literature. The squares are consistent with purely elastic behavior of the protein, the circles with viscoelastic behavior [24]. Specifically, 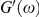 is constant in the elastic regime while it drops at low frequency in the viscoelastic regime (the low frequency drop is sometimes referred to as the Maxwell transition [24] and is the signature of viscoelasticity). (a) The storage modulus 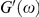 (the real part of the complex modulus, arbitrary unit) of the protein in the elastic (squares) and viscoelastic (circles) regimes, calculated from the data of Fig. 2 using eqs. 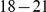. (b) The loss modulus 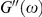 (the imaginary part of the complex modulus, arbitrary unit) of the protein 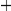 GNP system in the elastic (squares) and viscoelastic (circles) regimes, calculated as above. The real “storage/loss modulus” corresponding to 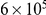 in the graph is 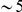 pN/nm.(TIF)

## References

[1] Zaccai G (2000). How soft is a protein? a protein dynamics force constant measured by neutron scattering.. Science.

[2] Frauenfelder H, Petsko GA, Tsernoglou D (1979). Temperature-dependent x-ray diffraction as a probe of protein structural dynamics.. Nature.

[3] Evans E, Ritchie K (1997). Dynamic strength of molecular adhesion bonds.. Biophys J.

[4] Merkel R, Nassoy P, Leung A, Ritchie K, Evans E (1999). Energy landscapes of receptor-ligand bonds explored with dynamic force spectroscopy.. Nature.

[5] Rief M, Gautel M, Oesterhelt F, Fernandez JM, Gaub HE (1997). Reversible unfolding of individual titin immunoglobulin domains by AFM.. Science.

[6] Rief M, Pascual J, Saraste M, Gaub HE (1999). Single molecule force spectroscopy of spectrin repeats: low unfolding forces in helix bundles.. Journal Molecular Biology.

[7] Cui Y, Bustamante C (2000). Pulling a single chromatin fiber reveals the forces that maintain its higher-order structure.. Proc Natl Acad Sci.

[8] Yang G, Cecconi C, Baase WA, Vetter IR, Breyer WA (2000). Solid-state synthesis and mechanical unfolding of polymers of t4 lysozyme.. Proc Natl Acad Sci.

[9] Radmacher M, Fritz M, Cleveland JP, Walters DA, Hansma PK (1994). Imaging adhesion forces and elasticity of lysozyme adsorbed on mica with the atomic force microscope.. Langmuir.

[10] Kawakami M, Byrne K, Brockwell DJ, Radford SE, Smith DA (2006). Viscoelastic study of the mechanical unfolding of a protein by afm.. Biophysical Journal.

[11] Kawakami M, Byrne K, Khatri B, Mcleish TCB, Radford SE (2004). Viscoelastic properties of single polysaccharide molecules determined by analysis of thermally driven oscillations of an atomic force microscope cantilever.. Langmuir.

[12] Kawakami M, Byrne K, Khatri BS, Mcleish TCB, Radford SE (2005). Viscoelastic measurements of single molecules on a millisecond time scale by magnetically driven oscillation of an atomic force microscope cantilever.. Langmuir.

[13] Wang Y, Zocchi G (2010). Elasticity of globular proteins measured from the ac susceptibility.. Physical Review Letters.

[14] Wang Y, Zocchi G (2011). The folded protein as a viscoelastic solid.. EPL.

[15] Singh-Zocchi M, Dixit S, Ivanov V, Zocchi G (2003). Single-molecule detection of dna hybridization.. Proc Natl Acad Sci.

[16] Tseng CY, Wang A, Zocchi G (2010). Mechano-chemistry of the enzyme guanylate kinase.. Europhysics Letters.

[17] Zocchi G (2009). Controlling proteins through molecular springs.. Annual Review of Biophysics.

[18] Sacquin-Mora S, Delalande O, Baaden M (2010). Functional modes and residue exibility control the anisotropic response of guanylate kinase to mechanical stress.. Biophysical Journal.

[19] Sekulic N, Shuvalova L, Spangenberg O, Konrad M, Lavie A (2002). Structural characterization of the closed conformation of mouse guanylate kinase.. Journal Biological Chemistry.

[20] Blaszczyk J, Li Y, Yan H, Ji X (2001). Crystal structure of unligated guanylate kinase from yeast reveals gmp-induced conformational changes.. Journal Molecular Biology.

[21] Choi B, Zocchi G (2007). Guanylate kinase, induced fit, and the allosteric spring probe.. Biophysical Journal.

[22] Wang Y, Wang A, Qu H, Zocchi G (2009). Protein-dna chimeras: synthesis of two-arm chimeras and non-mechanical effects of the dna spring.. Journal of Physics: Condensed Matter.

[23] Delalande O, Sacquin-Mora S, Baaden M (2011). Enzyme closure and nucleotide binding structurally lock guanylate kinase.. Biophysical Journal.

[24] Deshpande AP, Krishnan JM, Kumar PBS (2010). Rheology of Complex Fluids.. New York: Springer.

[25] Weltmann RN, Green H (1943). Rheological properties of colloidal solutions, pigment suspensions, and oil mixtures.. J Appl Phys.

[26] Kholodenko AL, Douglas JF (1995). Generalized stokes-einstein equation for spherical particle suspensions.. Physical Review E.

[27] Levine AJ, Lubensky TC (2000). One- and two-particle microrheology.. Physical Review Letters.

[28] Levine AJ, Lubensky TC (2001). Response function of a sphere in a viscoelastic two-uid medium.. Physical Review E.

[29] Go N, Noguti T, Nishikawa T (1983). Dynamics of a small globular protein in terms of low-frequency vibrational modes.. Proc Natl Acad Sci.

[30] Tseng CY, Wang A, Zocchi G, Rolih B, Levine AJ (2009). Elastic energy of protein-dna chimeras.. Phys Rev E.

[31] Hopfield JJ (1973). Relation between structure, co-operativity and spectra in a model of hemoglobin action.. Journal Molecular Biology.

[32] Russel WB, Saville DA, Schowalter WR (1989). Colloidal Dispersions.. Cambridge: Cambridge University Press,.

[33] Nuzzo RG, Fusco FA, Allara DL (1987). Spontaneously organized molecular assemblies. 3. preparation and properties of solution adsorbed monolayers of organic disulfides on gold surfaces.. Journal American Chemical Society.

[34] Taton TA (2002). Preparation of Gold Nanoparticle-DNA Conjugates.. http://dx.doi.org/10.1002s0471142700.

