# Supplementary figures and images for: Viscoelastic Transition and Yield Strain of the Folded Protein
Source: PLoS One. 2011 Dec 8;6(12):e28097. doi: 10.1371/journal.pone.0028097 (PMC3234265; doi:10.1371/journal.pone.0028097)

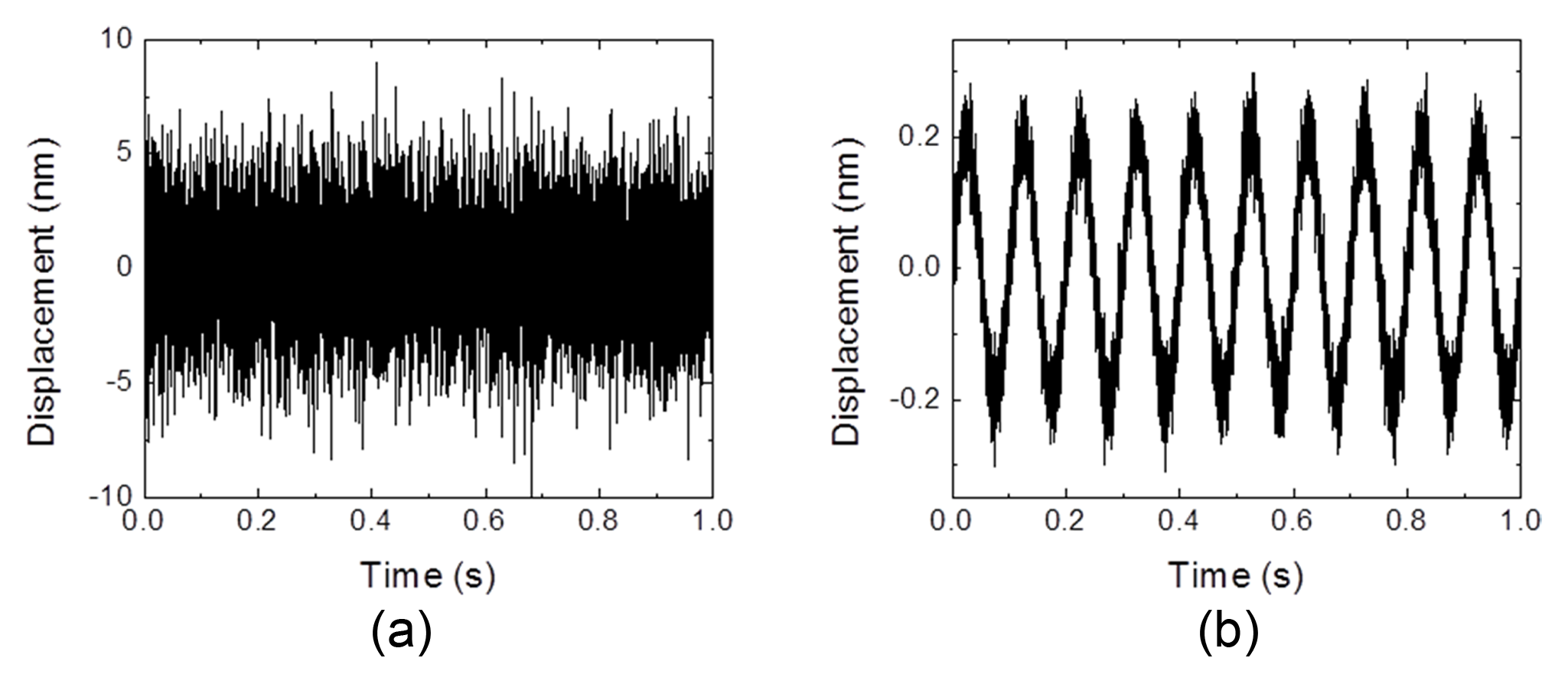

Supplement: Figure S1 — Thermal fluctuations are averaged out by measuring over many GNPs: case of an elastic tether. Simulated displacement (a) of a single GNP; (b) averaged over 5000 GNPs, attached to elastic springs in the presence of thermal noise. We ran numerical simulations and looked at the displacement of GNPs attached to an elastic spring when a sinusoidal external force is applied. The equation of motion of the gold nanoparticle, including thermal fluctuations, is where is the displacement of the GNP, the hydrodynamic dissipation coefficient of the GNP, the spring constant, the applied external force: , where pN and the alternating frequency Hz, and a stochastic force (the Brownian motion term) satisfying the following two relations: and . The parameters of the simulation were directly from experimental measurements [14], [30]: pN/nm, kg/s. We note that the applied force in the experiments is pN. In the presence of the Brownian noise term, the displacement of a single GNP is dominated by thermal fluctuations. However, the average displacement over GNPs is not: the average displacement oscillates at the same frequency as the applied force. The simulation also shows that by averaging over many particles, it is not impossible to measure “very small” displacements (sub-Angstrom, at high frequencies, compared to thermal fluctuations of the individual GNPs are nm) buried in large thermal noise. (TIF) [file pone.0028097.s001.tif]

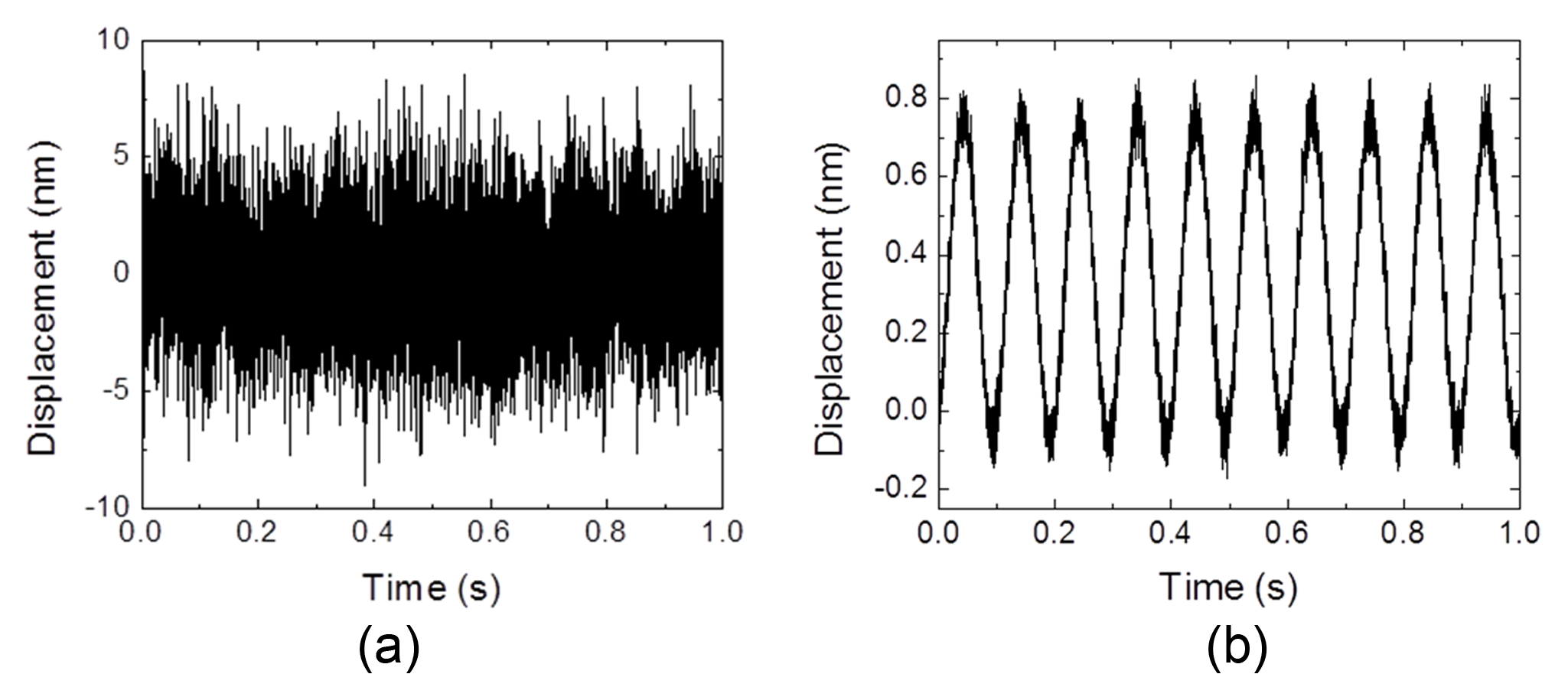

Supplement: Figure S2 — Thermal fluctuations are averaged out by measuring over many GNPs: case of a viscoelastic tether. Simulated displacement (a) of a single GNP; (b) averaged over 5000 GNPs, attached to viscoelastic Maxwell elements (eq. 5) in the presence of thermal noise. We ran numerical simulations similar to the ones in Fig. S1 but with a slightly different equation of motion for the gold nanoparticle: where is the force from the Maxwell element (eq. 5). The parameters were again directly from experimental measurements [14], [30]: pN/nm, kg/s, kg/s. Similar to Fig. S1, in the presence of the Brownian noise term, the displacement of a single GNP is dominated by thermal fluctuations. However, the average displacement over GNPs is not: the average displacement oscillates at the same frequency as the applied force. The simulation also shows that by averaging over many particles, it is not impossible to measure “very small” displacements buried in large thermal noise. (TIF) [file pone.0028097.s002.tif]

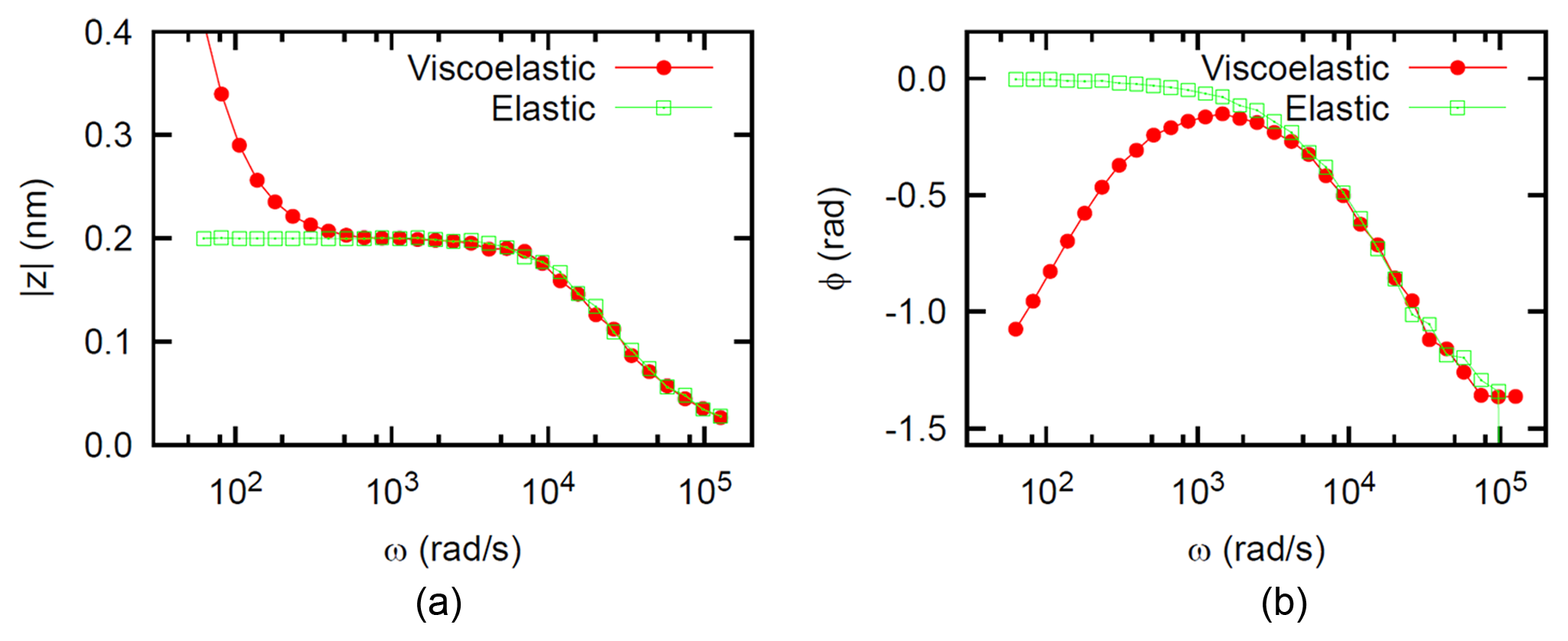

Supplement: Figure S3 — Simulated frequency response in the presence of thermal noise. Fitting the average displacement (Fig. S1b and Fig. S2b) with a sine wave gives the amplitude and phase (the quantities we measure in the experiments). By sweeping the driving frequency () over a range, we obtain numerically the frequency response (a: amplitude; b: phase) in the presence of thermal noise, which are exactly the same as the analytical results from eq. (4), without the Brownian motion term. Thus the present measurement method is able to average out thermal noise. (TIF) [file pone.0028097.s003.tif]

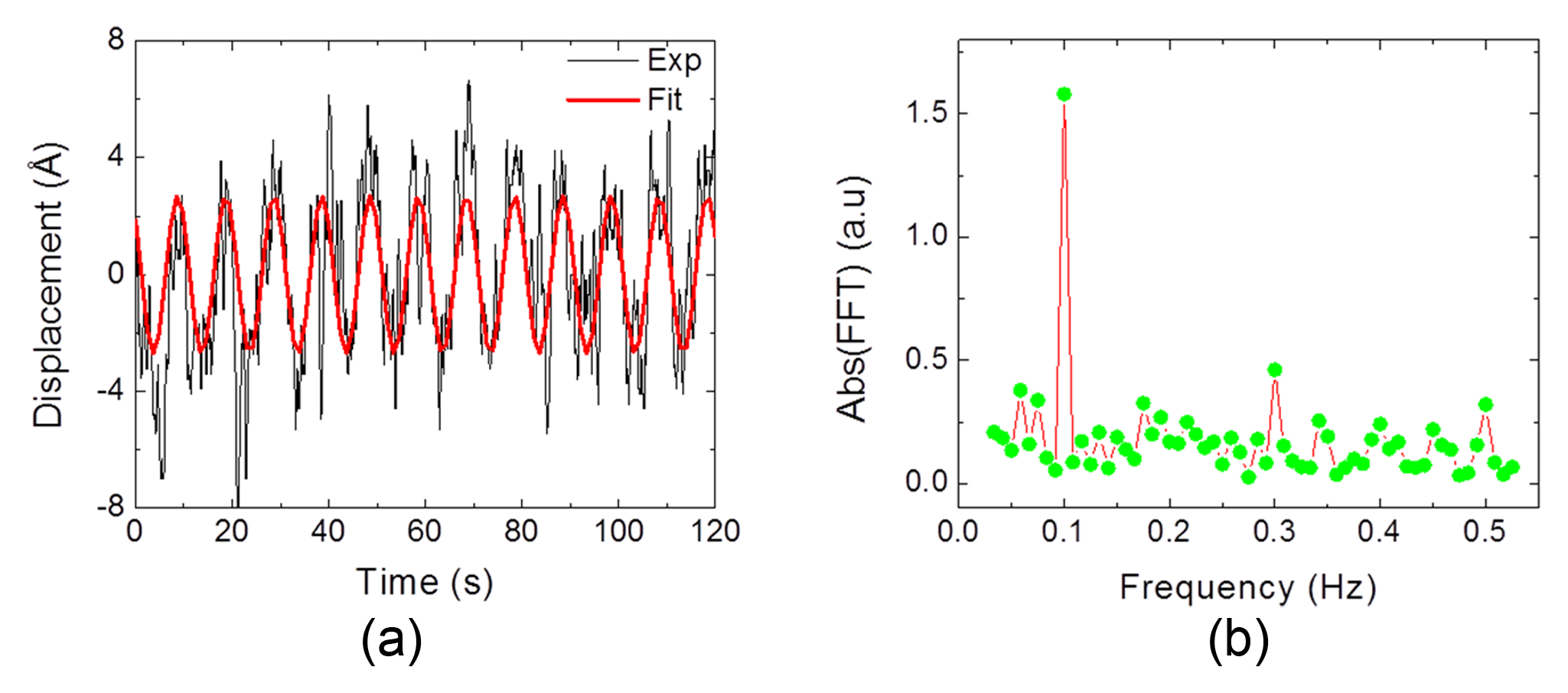

Supplement: Figure S4 — AC susceptibility of a single stranded DNA (experimental measurements). In addition to numerical simulations, we also show experimentally that the average displacement of many gold nanoparticles is sinusoidal with the same frequency as the driving force. In order to observe directly the oscillation of the average displacement, a single stranded DNA coil [13], [14], which is softer than a globular protein and has larger deformation, has been chosen; and also the driving frequency is low: 0.1 Hz. (a) The instantaneous displacement (thin black curve) averaged over GNPs and a fit with a sine wave (thick red curve) giving a fitted frequency of 0.1 Hz, which is the same as the driving frequency. (b) Fourier transform (FFT) performed on the instantaneous displacement. An obvious peak is present at 0.1 Hz. (TIF) [file pone.0028097.s004.tif]

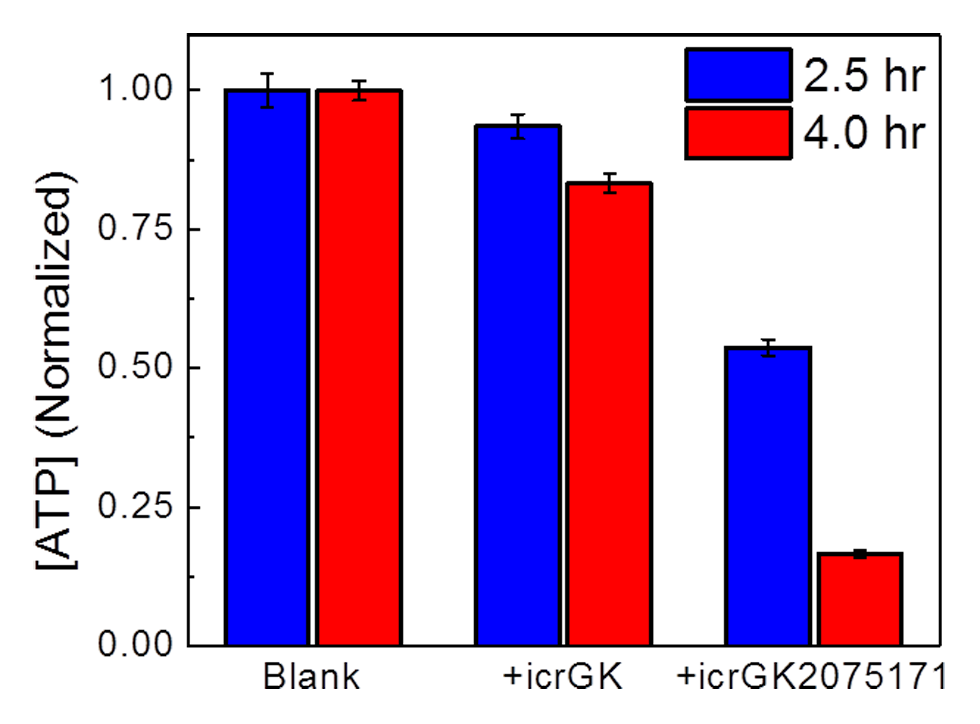

Supplement: Figure S5 — Enzymatic Activity of Immobilized Proteins. We are interested in the mechanical properties of the folded protein. It is then essential to make sure that the proteins immobilized on the gold surfaces are folded and functional. We measured the enzymatic activity of GK attached to the Gold-coated slide using the Kinase-Glo (Promega, Madison, WI) luminescent assay. The assay quantifies the depletion of ATP following the kinase reaction: GMP+ATPGDP+ADP. The assay reagents rely on the properties of a proprietary thermostable luciferase that is formulated to generate a stable “glow-type” luminescent signal which is produced by the luciferase reaction. The intensity of the generated luminescence is directly proportional to the amount (or concentration in a fixed volume) of ATP in the solution (see manufacturer manual). A mixture of ATP and GMP solution at optimized concentrations is added on the surface with the immobilized proteins. The kinase reaction is incubated for 2.5 or 4 hours. Then the solution is removed, mixed with the luminescence assay reagents and incubated for 10 minutes. Luminescence was measured with a DTX 800 multimode detector (Beckman Coulter). The figure shows the concentration of ATP remaining after the specified time for specifically immobilized guanylate kinase (+icrGK2075171: the mutant of this study, with Cys residues substituted at positions 75 and 171) and two controls: without proteins on the slide (Blank) and with nonspecifically bound GK (+icrGK: this mutant has no Cysteins). The result shows that the specifically immobilized Guanylate Kinase is functional and therefore folded on the gold surface. (TIF) [file pone.0028097.s005.tif]

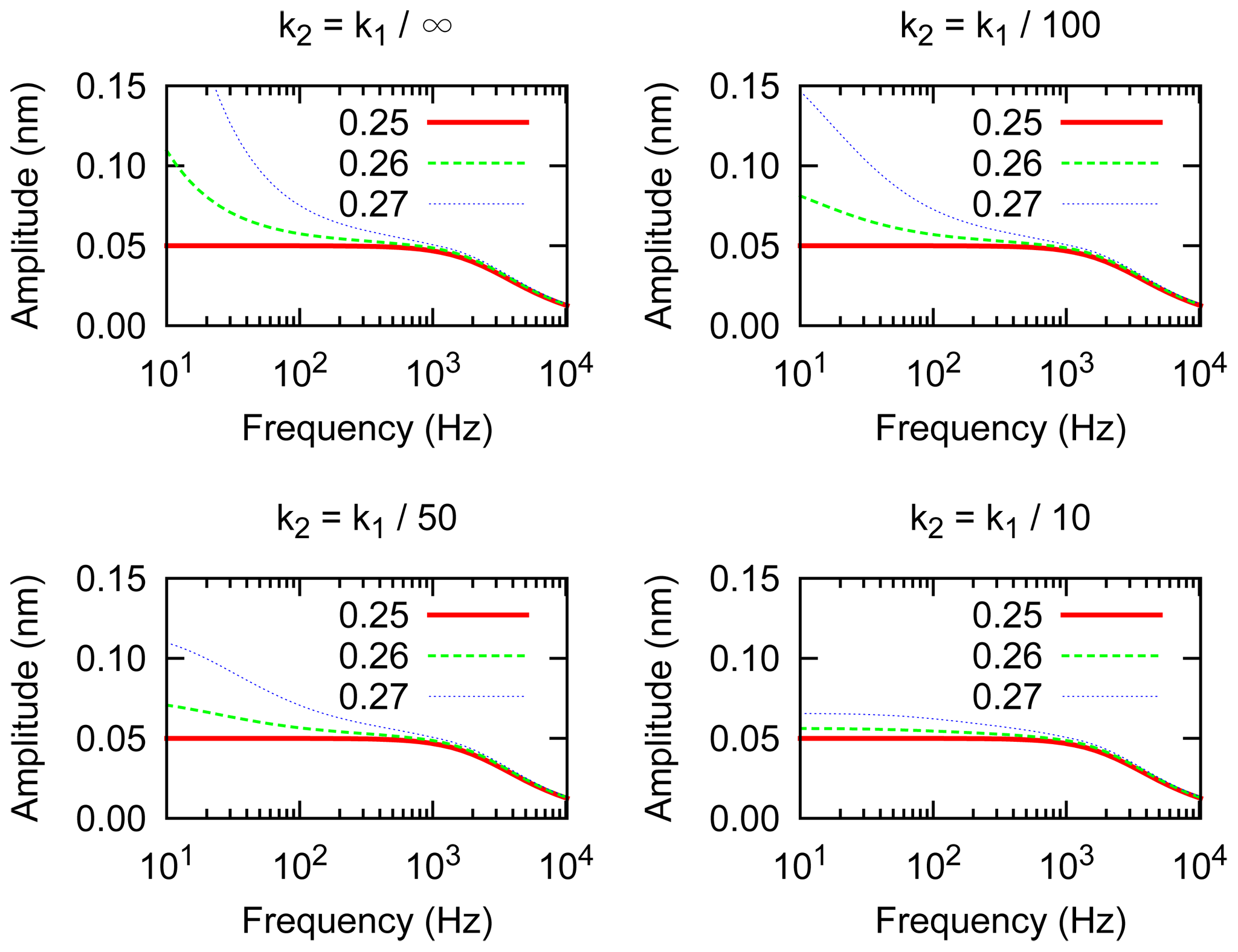

Supplement: Figure S6 — Numerical simulation of nonlinear springs. We computed numerically the frequency response of a nonlinear spring, with a force-extension curve: if , or if , or if , attached to a “bead” characterized by a hydrodynamic dissipation coefficient (eq. 4). In eq. (25), represent the two slopes of Fig. 3 and is the “yield deformation”. We ran the simulation using the measured values for the three parameters: 0.5 Å (Fig. 2), kg/s, 5 pN/nm [13], [30], and varying and the amplitude of the forcing. The figure shows the results for the amplitude of the response, for 4 different values of (and thus the ratio ; is fixed at the value of the elastic spring constant of the protein) under different driving forces: 0.25, 0.26 and 0.27 pN. The figure shows that one can obtain a response similar to the inset in Fig. 2, but only for very large ratios of the slopes . In contrast, the experimentally measured ratio (Fig. 3) is , and for such “nonlinear springs” the response always looks like the last graph, quantitatively and qualitatively different from Fig. 2. Nonlinear springs with small 's show Maxwell-type response. But a nonlinear spring with large does not. (TIF) [file pone.0028097.s006.tif]

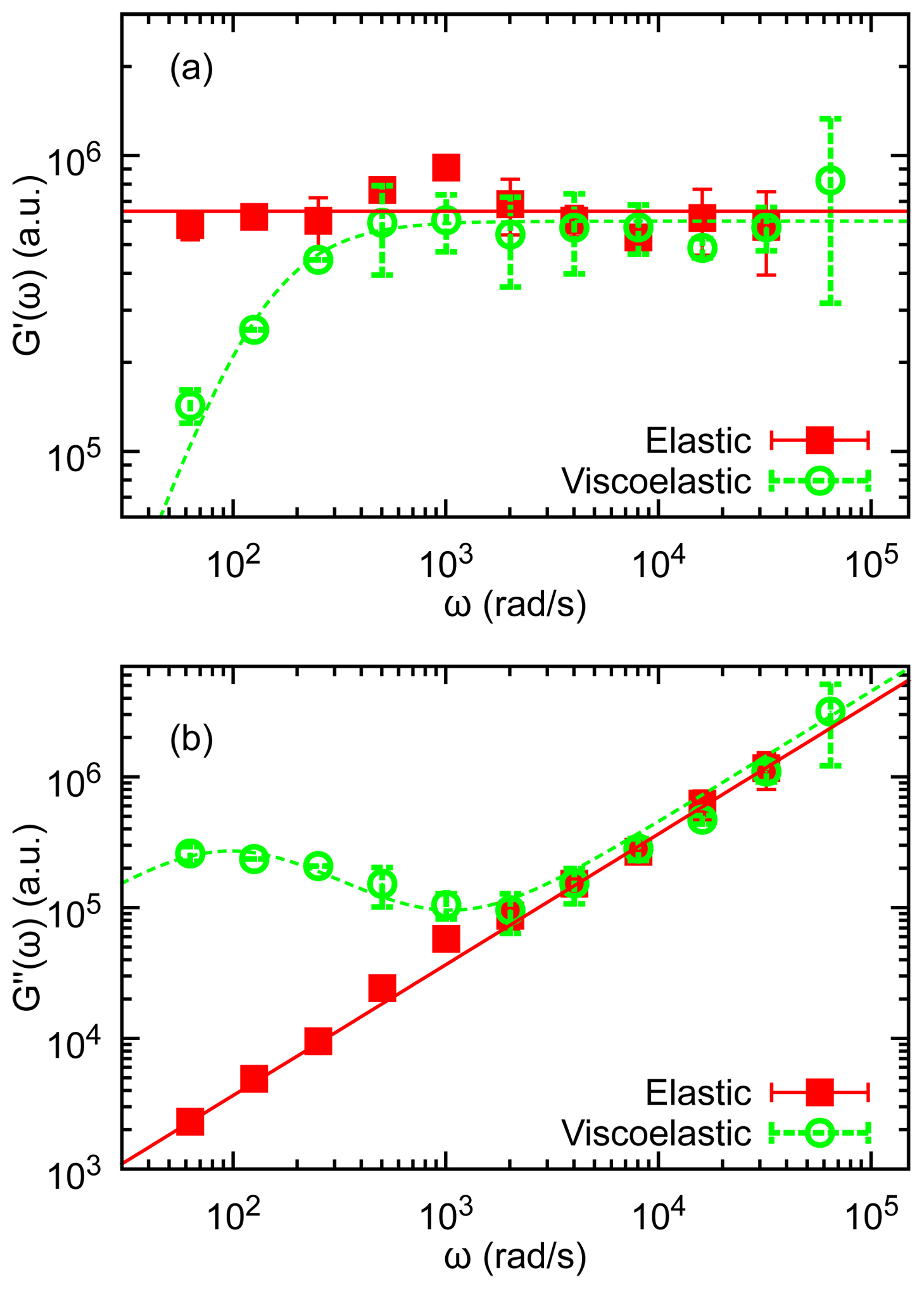

Supplement: Figure S7 — Calculated real and imaginary parts of the complex modulus. We represent the same data and fits of Fig. 2 in terms of storage modulus and loss modulus calculated from (eqs. ), since this is the canonical representation in the rheological literature. The squares are consistent with purely elastic behavior of the protein, the circles with viscoelastic behavior [24]. Specifically, is constant in the elastic regime while it drops at low frequency in the viscoelastic regime (the low frequency drop is sometimes referred to as the Maxwell transition [24] and is the signature of viscoelasticity). (a) The storage modulus (the real part of the complex modulus, arbitrary unit) of the protein in the elastic (squares) and viscoelastic (circles) regimes, calculated from the data of Fig. 2 using eqs. . (b) The loss modulus (the imaginary part of the complex modulus, arbitrary unit) of the protein GNP system in the elastic (squares) and viscoelastic (circles) regimes, calculated as above. The real “storage/loss modulus” corresponding to in the graph is pN/nm. (TIF) [file pone.0028097.s007.tif]
